# Supplementary material for: The larval Drosophila mushroom body balances lateralized sensing and interhemispheric integration
Source: bioRxiv. 2026 Feb 3:2025.03.02.641007. Preprint. [Version 2] doi: 10.1101/2025.03.02.641007 (PMC12889419; doi:10.1101/2025.03.02.641007)
Supplement: Supplementary file 1 [file media-1.pdf]

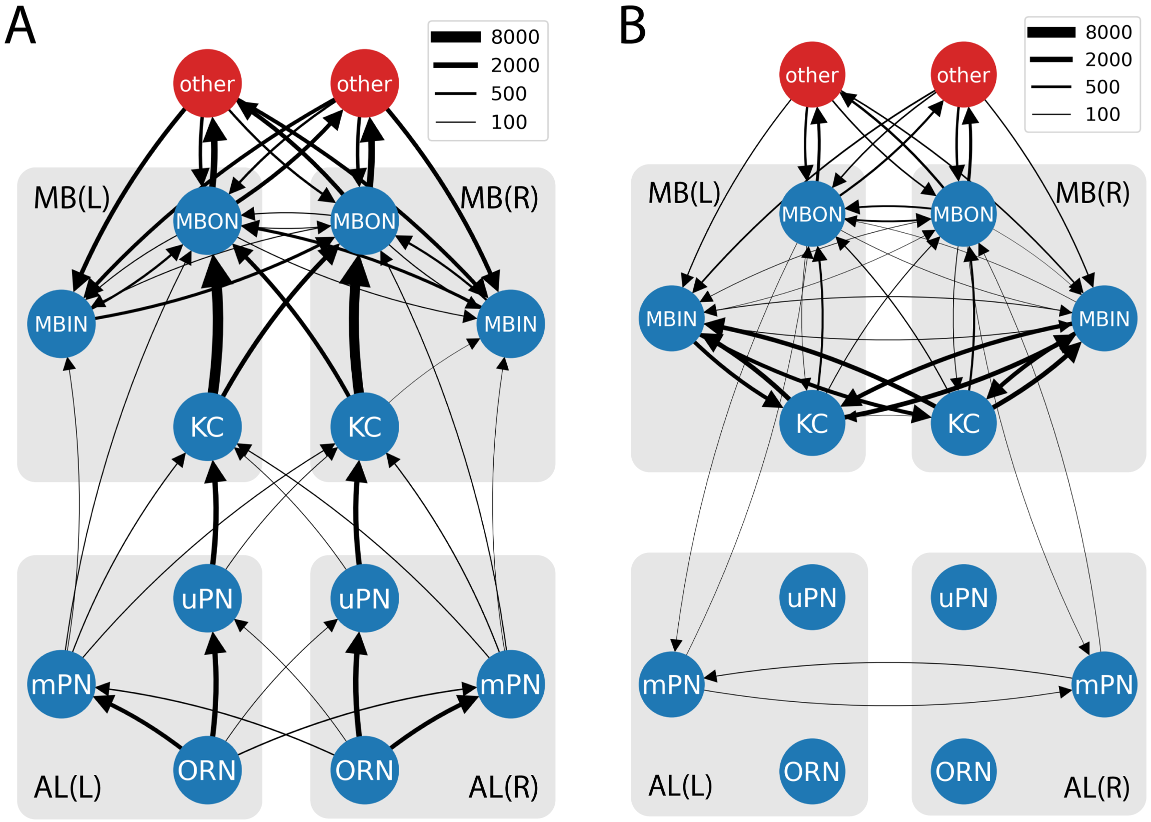

Figure S1: Axo-dendritic and axo-axonic connectivity of the larval olfactory system. Summarized wiring diagrams of the L1 larval olfactory system, analogous to Fig. 1A except that only axo-dendritic (A) or axo-axonic (B) connectivity is shown. Data from Winding *et al.* (*1*). Related to Fig. 1.

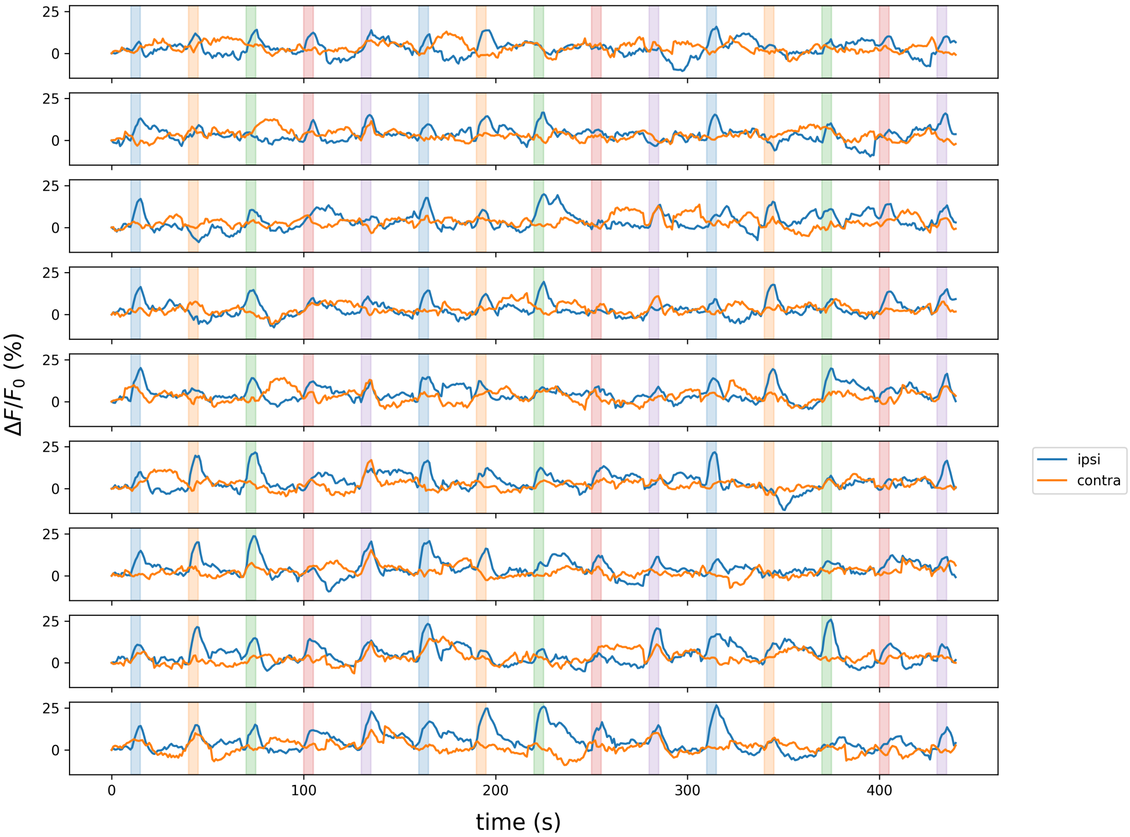

Figure S2: Raw calcium responses of ipsi- and contralateral KCs to unilateral olfactory stimulation. Full calcium response traces for each animal from Fig. 1E–H. Each row represents a different larva ($n = 3$ technical replicates from each of 9 animals). The side ablated (left or right) was randomized across animals. Shaded bars indicate odor periods. Color code: MePhS, methyl phenyl sulfide (blue); EtOAc, ethyl acetate (orange); BzCHO, benzaldehyde (green); GeOAc, geranyl acetate (red); 1PnOH, 1-pentanol (violet). All odors were used at 10^{-4} dilution. Related to Fig. 1.

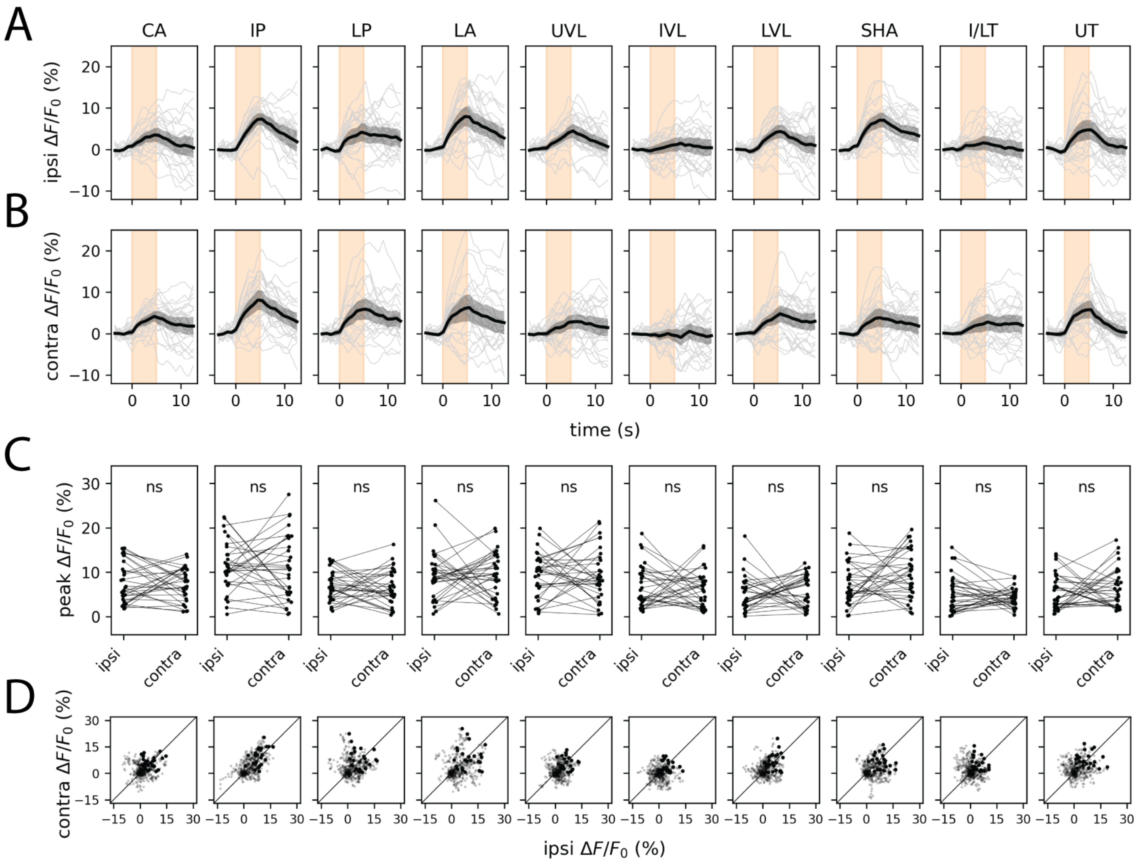

Figure S3: Symmetrization of unilateral olfactory stimuli by the MBIN ensemble (alternate odorant). Plots show ipsilateral (A) and contralateral (B) presynaptic GCaMP6s transients and peak ipsilateral vs. contralateral response amplitudes (C) evoked by one-sided stimulation with ethyl acetate (10^{-4}). In A and B, dark lines represent grand averages over $n = 8$ animals and 4 stimulus repetitions per animal; gray shaded regions are bootstrapped 95% CIs. Colored vertical bars represent 5 s stimulus periods. D. Scatter plots of ipsilateral and contralateral MBIN activity in the 15 s following stimulus onset. Bold points represent peak responses. Anatomical abbreviations: CA, calyx; IP, intermediate pendunclе; LP, lower peduncle; LA, lateral appendix; UVL, upper vertical lobe; IVL, intermediate vertical lobe; LVL, lower vertical lobe; SHA, shaft; I/LT, intermediate/lower toe; UT, upper toe. Statistics: n.s., $p_{\text{adj}} \geq 0.05$ (paired Student's t-test with Holm's correction). Related to Fig. 2.

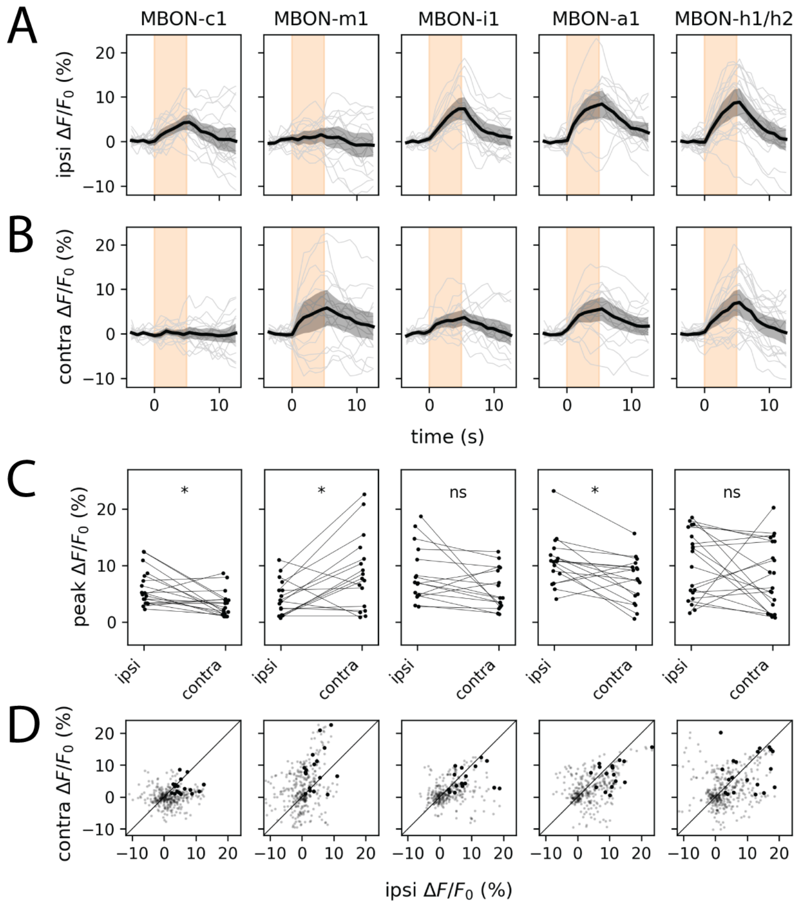

Figure S4: MBONs exhibit diverse ipsi- vs contralateral tuning to asymmetric odor stimuli (alternate odorant). Plots show ipsilateral (A) and contralateral (B) presynaptic GCaMP6s transients and peak ipsilateral vs. contralateral response amplitudes (C) evoked by one-sided stimulation with ethyl acetate (10^{-4}). In A and B, dark lines represent grand averages over $n = 7 \pm 1$ animals and 3 stimulus repetitions per animal; gray shaded regions are bootstrapped 95% CIs. Colored vertical bars represent 5 s stimulus periods. D. Scatter plots of ipsilateral and contralateral MBIN activity in the 15 s following stimulus onset. Bold points represent peak responses. Diagonal lines represent equality between ipsilateral and contralateral response amplitudes. Each column shows responses in a specific MBON type, labeled by the corresponding split-GAL4 line (see Methods). Statistics: n.s., $p_{\text{adj}} \geq 0.05$; *, $p_{\text{adj}} < 0.05$; **, $p_{\text{adj}} < 0.01$ (paired Student's t-test with Holm's correction). Related to Fig. 3.

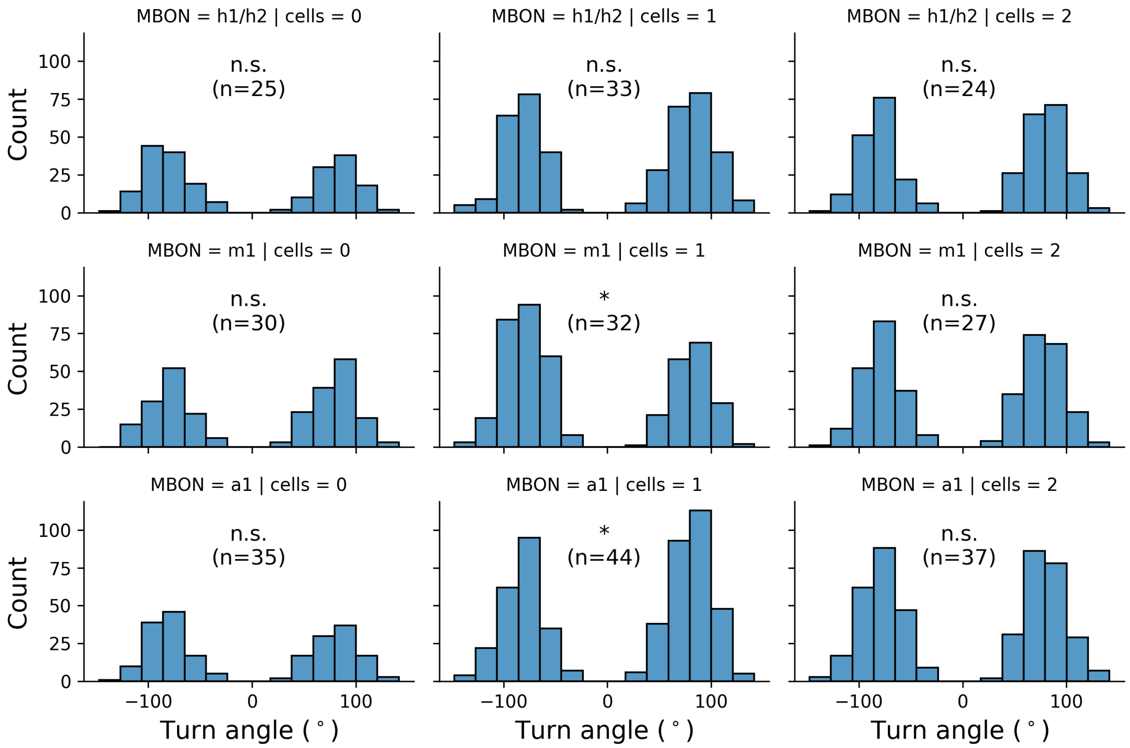

Figure S5: Optogenetic activation of individual MBONs modulates turning in a variably side-biased manner. Histograms of turn angle (pooled over all detected turns by all animals within each class) triggered by optogenetic stimulation of 0, 1, or 2 cells (columns) of the indicated MBON type (rows). For the 2-cell case of MBON-h1/h2, animals expressed the optogenetic effector CsChrimson::mVenus in 1 cell of each hemisphere. Statistics: n.s., $p_{\mathrm{adj}} \geq 0.05$; *, $p_{\mathrm{adj}} < 0.05$ (Wilcoxon signed-rank test with Bonferroni correction). Related to Fig. 4.
